# Supplementary material for: Monitoring the emotional facial reactions of individuals with antisocial personality disorder during the retrieval of self-defining memories
Source: PLoS One. 2022 Jun 8;17(6):e0268818. doi: 10.1371/journal.pone.0268818 (PMC9176833; doi:10.1371/journal.pone.0268818)
Supplement: Attachment — Submitted filename: review..docx [file pone.0268818.s002.docx]

Thank you for inviting me to review this manuscript which presents a study that examines the facial reactivity during the retrieval of self-defining memories in individuals with Antisocial Personality Disorder (APD) and controls. The study has some interesting findings and used a new methodology to access participants reactions (Facereader).

However there are a number of weaknesses that affect my enthusiasm for the manuscript that are outlined below with some suggestions on how to address these:

1) Overall the paper would be improved by having some-one proofread it and improve the flow to make it easy to read and follow.
2) Title:
   a. I would suggest changing to: “Facial reactions to retrieving self-definying memories.. or monitoring facial reactivity while asked to retrieve…”
3) Abstract:
   a. The first sentence in the introduction is awkward. What do the authors mean “very few studies have investigated its production”. Are the authors referring to naturally displaying facial expressions/producing facial expressions? They should also be specific. There are a number of studies examining facial reactivity/expressivity..
   b. Similarly in the abstract “during retrieval of autobiographical memories to be as close as possible to their personal feelings?” This is not clear and should be better phrased.
   c. When the authors say they showed less neutral facial expression, does this mean they were more expressive? Again not clear. Also relates to later comment in my review.
   d. Abbreviations (ASPD) in abstract need to be defined.
   e. The authors should be careful in their interpretation of the findings stating that “this study highlights the core role of anger in this psychopathology” –this is very general, the sample included individuals with APD so findings should be limited to APD.
4) Introduction
   a. P.4 There is a problem with authors argumentation. The first sentence argues that most studies in the area do not have ecological validity, and that their study examines spontaneous facial expression that arise when participants are asked to think of their emotional experiences. The problem with this is that the task they used had limitations which are outline below (next comment) and these are not mentioned in the limitations section..
5) Method
   a. There is a problem with the design on the task used. The authors used vague instructions asking participants to recall emotional events (which they then coded as positive, negative, mixed or neutral) vs asking them to recall a negative/positive/neutral memory. Looking at the data it is not clear that the task actually worked – did the participants actually follow the instructions or failed to do so due to bad memory/suppressed memory or simply because they did not want to understand or want to comply with instructions. This also raises concerns as figure 3 suggest that the task of recalling emotional events did not have the expected reaction for control participants (increase in happy expressions with the recall of positive events, or increase of sad angry or fearful facial expression in the event that a negative memory was recalled). This might be because of the high level of neutral expressions recorded by Facereader which is related to my next comment and suggestion about removing neutral expressions from the analysis.

6) Results
   a. How original is this study and the findings presented? It is not surprising that individuals with APD are angry. Individuals with APD that end up in forensic settings obviously have anger issues. The data focused on fear are unexpected and interesting..
   b. The authors do not explain the findings well or in a context that the reader that is not familiar with how Facereader software operates, can be understood. The software by default categorizes the facial expression to neutral if it can not classify it as any other emotion. So what does it mean in relation the authors findings? It might be worth removing the neutral data and focus on the other emotions (happy, fear, sad, angry) to pick up any differences. It is hard to understand if the task participants were asked to performed “worked” as even when the memories were categorized as positive/negative the main expression produced was neutral. By removing the neutral data the authors might be able to pick up on more interesting findings.
   c. Figures 1-3 are not clear (font is small and fuzzy) making the figures hard to interpret. A suggestion would be to use color so it easier to differentiate between the emotions if this is not possible to organize them in a way that helps the reader (lighter color neutral, darker greys negatives then positives or something like that).
   d. Also there seems to be a large deviation between the control group and the APD group with regard to age and years of education. Were these looked into?
   e. Minor point but table 2 and figures have different font than the rest of the document.
7) The discussion should be more focus on the explaining how the findings of the current study fit in the literature. Main finding seems to be that APD are angry but this is not new..
