# Supplementary material for: Monitoring the emotional facial reactions of individuals with antisocial personality disorder during the retrieval of self-defining memories
Source: PLoS One. 2022 Jun 8;17(6):e0268818. doi: 10.1371/journal.pone.0268818 (PMC9176833; doi:10.1371/journal.pone.0268818)
Supplement: Attachment — Submitted filename: Review.docx [file pone.0268818.s003.docx]

**Review**

The current manuscript describes a study that investigates the expression of facial emotions during the retrieval of self-defining memories in participants with ASPD compared to a control group. The study includes an interesting sample with ASPD diagnosis and includes an assessment of the PCL-R. Further, they used an innovative method (FaceReader) to detect the expressed emotions. Despite these advantages and the generally interesting idea of the study, I have some concerns and open questions that should be addressed. My main concerns are associated with the theoretical background and the implications of the results. Please see my detailed comments below.

Introduction

- In my opinion, it would be more accurate to state that, to date, research has focused on the ability to recognize emotional facial expressions but not expressing them because I think there are more than few studies investigating the detection of FE in other individuals in participants with antisocial personality disorders. Of course, many of the existing studies did not investigate participants with a diagnosis of ASPD (which is a clear advantage of the current study) but instead antisocial or psychopathic personality traits/offenders/etc. However, I would like this statement of the authors to be more specific, especially since the authors do not only cite papers that focus on a sample with a diagnosis of ASPD.
- **Second, I think the manuscript would benefit from a stronger differentiation between expressing emotions (e.g., anger), which can be objectively measured, and subjectively experiencing emotions.**

**In this context, I would like to ask, why the authors did not assess a subjective description of what the participants felt. Especially, since the instruction for SDM included choosing memories that elicit strong feelings and the use of the FaceReader instead of electromyography allows distinguishing between the different negative emotions.**

- **Further, I would like to suggest providing a more detailed explanation why particularly the expression of emotions is relevant in individuals with ASPD.**
- I would like to ask for a definition of “ecological setting”.
- As psychopathy and its factors/facets are analyzed, in my opinion, the construct of psychopathy and the PCL-R should be described in more detail.
- In my view, the manuscript would benefit from a clear statement with regard to the hypotheses. (Exploratory analyses should be labeled as such if necessary.)

Methods

- How was ensured that that the control participants had no mental or behavioral difficulties? How were they recruited?
- It seems like education level and age might differ between groups. I think the authors should comment on that in their manuscript.
- How was the neutral video fragment for calibration selected?

Results

- What was the minimum number of SDM per participant after the exclusion?
- At first, I was confused by the structure of the statistical analysis section and the results. In my opinion, the results regarding the valence of the SDM and the associated chi squared test should be reported in the beginning, because this is essential for the interpretation of the results (the test is mentioned in the second paragraph of the methods but the results are reported in the third paragraph of the results section).
- Figure 1: Information regarding the boxplots might help the reader to better understand the results (e.g., does the boxplot show the mean or the median?)
- I would be interested in the estimate for the main effect of valence in Model 2.
- I think figure 3 is not referenced in the text.
- I understand that in Table 1 shows only the interaction effects for model 4 but not the estimates that are reported for model 3. Why? Are there differences?
- The naming of the factors and subscales of the PCL-R should be consistent in the table and the text.

Discussion

- I think the conclusion that participants with ASPD used fewer FE associated with the neutral state than the control participants independent of the valence of the SDM should not be based on model 1, since this model did not include the valence.
- The discussion of the results with regard to psychopathy is mainly focused on the facet 3 and 4. However, in the introduction it seemed like an aim of the study to investigate if experiencing fewer emotions is related to callous-unemotional traits, which should also be represented by facet 2.
- **I think the limitation that the degree of congruence between FE and subjectively experienced feelings was not measured is a major limitation and I think the manuscript would benefit from a more detailed discussion of this issue and its relevance for the conclusion than can be drawn based on this study.**
- **Finally, I am wondering if the stronger expression of anger during retrieval of SDM in individuals with ASPD can be generalized to everyday situations and can be interpreted as chronic anger. This concern is strengthened by the fact that SDM in individuals with ASPD might be more traumatic and anger evoking that the SDM of control participants. Since only the general valence of the SDM was considered but not the related emotion or the intensity, in my view, it might still be possible that group differences in anger expression are based on differences in SDM.**
