# Supplementary material for: Monitoring the emotional facial reactions of individuals with antisocial personality disorder during the retrieval of self-defining memories
Source: PLoS One. 2022 Jun 8;17(6):e0268818. doi: 10.1371/journal.pone.0268818 (PMC9176833; doi:10.1371/journal.pone.0268818)
Supplement: Attachment — Submitted filename: Response to Reviewers nov2021.docx [file pone.0268818.s004.docx]

Dear Professor Eisenbarth

We wish to thank you for your interest in our study and we highly appreciate your suggestions that we should provide a new revised manuscript. We also want to thank the reviewer providing very useful comments.

Here are our responses to each reviewer’s concerns

Reviewer #1: 

**Introduction**
**Can the authors please define ASPD? What are the features, and how is it distinguished from psychopathy?**

Answer: We have amended the introduction and added a definition of ASPD. The following sentences have been added to the introduction: “Yet, ASPD are associated with emotional disturbance and these subjects are generally described as self-centered, impulsive and callous. They find it difficult to conform to laws or norms, lack empathy and concern for others and encounter difficulties in having a stable relationship [11].”

In order not to confuse the reader, we have chosen to add a paragraph on the links and distinctions between ASPD and psychopathy in the method section. The following sentences have been added to the methods at the Assessment of psychopathic traits section: “Individuals with ASPD may present varying levels of psychopathic traits ranging from totally absent to a full diagnosis of psychopathy. ASPD and psychopathy share much of their semiology and while every person with a psychopathic personality has an ASPD, the contrary is not true [32,33]. According to Venables et al. (2014), one of the main distinctions between these two personalities is the cold-heartedness component, which is a core characteristic of psychopathy [34]”

**Relatedly, the authors should make it clear in their literature review whether prior studies assessed ASPD vs. Psychopathy. For example, the authors cite Künecke et al. (2018) as a study of ASPD, yet the study instead assessed psychopathy. The distinction is important, as behavioral, physiological, and neural correlates of the two diagnostic categories often differ.**

Answer: We have corrected the sentence in the introduction section as follows: “Contrary to the findings of Fanti et al. [9], Künecke et al. [10] found that antisocial offenders with different levels of psychopathy assessed in German forensic-psychiatric hospitals and correctional facilities did not differ from control participants on their corrugator muscle response to the observation of the FE of others”.

**The authors note that FaceReader is less obtrusive than electromyography. But doesn’t FaceReader also pick up on contractions unrelated to expressions of emotion and nonverbal communications (p. 4 line 81-83)? Is this also a limitation of FaceReader?**

Answer: Yes it is right but the FaceReader unlike the EMG was created to detect emotional facial expressions in tasks such as speaking. Also our two groups were in exactly the same experimental situation so that speech movements which were detected in the same way in both groups. In addition we added the following sentence in the introduction: FaceReader is a non-invasive technology that requires the use of a video camera. It accounts for the activation of several emotional action units and for the joint activation of several facial muscles corresponding to a given emotional facial expression.

**The authors should provide a citation for the theory of six basic emotions (probably a paper by Paul Ekman). However, I’d also recommend discussing more recent data that contradict this theory (for example, Barrett, Adolphs, Martinez, Marsella, & Pollak, 2019). These data suggest that six basic emotions are not universally expressed and are not exact readouts of a person’s emotional state.**

Answer: Indeed, we have chosen to focus on Ekman's theory because it is on the basis of this conceptualization that the FaceReader was developed. However, we thank you for your comments that allow us to open the results to other models and methodologies for future research. We have added the following sentences at the end of the discussion: " Finally, we chose to focus the present study on the theory of basic emotions [28,60] using the dominant FE as indicator. However, other studies propose a more functional conceptualization of emotions more adapted to studying pathological expression of emotions [50,61]. In future studies, it would be interesting to use a more dimensional methodology to further examine the production of facial expressions during the recall of autobiographical memories. We suggest a more precise analysis of the simultaneous activation of the various action units associated with emotions during the recall of memories”.

**Did the authors describe all their hypotheses in the introduction? I only see a hypothesis about increased anger expression during SDM retrieval in the ASPD group**

Answer: As you point out our hypothesis part was not clear, we decided to rewrite it in the following way:“ We hypothesize that individuals with ASPD produce more anger-associated FE than healthy participants during the retrieval of SDM. We also examined how participants with APSD expressed other emotions during memory recall (happy, sad and scared) in comparison with control participants, and quantified the part devoted to the non-expression of emotional FE (neutral states).

**Methods
There is not enough information about the control group. How was the control group recruited? Was the control group matched to the ASPD group? Was the control group assessed for ASPD or psychopathy. The control group seems to be younger and more educated than the ASPD group, and these differences do not seem to be controlled for in the analyses.**

Answer: We have added information on the recruitment of our control group. We have also added a sentence to explain why we were unable to match on education level. As you point out, there is an age difference between our two groups, however we ran models to see if the activation of the action units could be explained by age and none of the models ruled out the null hypothesis. We have added the following sentences to the Participant section of the Methods:“

The control sample was recruited through an advertisement distributed on social networks and from the staff of the universities of Lille in France and Mons in Belgium.

There was no significant age effect on the various facial expressions (neutral, happy, angry sad and scared) between the two groups. However, since most ASPD participants presented conduct disorders during childhood associated with school difficulties, we were not able to match education levels between APSD and control participants.

**Why was the SCID-II used to assess ASPD when the current version is the SCID-5?**

Answer: The commentary is justified but the SCID 5 has not yet been validated in French. We have kept the version used by the teams of psychologists in the departments where the patients were recruited and which corresponds to the DSM-IVR Axis 2 classification. Therefore, we added the following sentences in the Methods section, " Diagnostic scales (SCID II and PCL-R) were administered upon admission by trained professionals of the forensic hospital. Scores and diagnoses were extracted from the inpatients’ medical files."

**On p. 10 line 238-239, the authors lay out the rationale for their analytic strategy: “Bayesian analyses corresponded to the best statistical method in view of the sample size and the data of the experimental paradigm” What does this mean? Why, specifically, was this the best method?**

Answer: As we performed our statistical analyses with R software and also because of our sample size and the methodological design of our research, the Bayesian method was the most efficient. We added the following sentences to the statistical analysis part of our method:“ Beta distribution was used since values ranged from 0 to 1. Mixed-effects regressions were the most suitable as data were not independent and each participant retrieved several SDM. Bayesian analyses were the best statistical method considering sample size and data of the experimental paradigm.”

**Why were mixed effects Bayesian models not used for the PCL-R analyses? Why were PCL-R Total scores not analyzed?**

Answer: Thank you for your questions about our analysis of the PCL-R. Following your feedbacks, we have decided to delete these analyses, as we question the statistical power of these results given the size of our ASPD sample. In addition, we believe that this part led to confusion in the concepts (ASPD, psychopathy) for the reader.

**Were corrections for multiple comparisons performed?**

Answer: As we did our analyses via the Bayesian method we did not have to make this correction.

**Results
I don’t see any description of the types of self-defining memories each group reported. This seems like an important detail. Did the ASPD group report more negative events than the control group? Did they report more intense events than the control group? Did participants provide subjective ratings of their emotional state? My main question: did the group differences arise simply because the ASPD group reported more negative life events (which would likely result in more negative and angry expressions while participants described the events)?**

Answer: We have added a descriptive part about the type of SDM recalled in our two populations. However, we made a mistake and did not place it in the right paragraph, so it was not easily readable. There is no difference between our two populations regarding the type of memories recalled. The following sentences are now available in the results:“ In individuals with ASPD, 34.7% of the SDM were categorized as neutral, 22.8% as positive, 21.8 % as negative and 20.8% as mixed (χ^2^=2.30, *p*=.513). In control participants, 16.2% of the SDM were categorized as neutral, 36.2% as positive, 22.9% as negative and 24.8% as mixed (χ^2^=4.23, *p*=.238). Chi-squared tests demonstrated a difference between the two groups (χ^2^=10.46, *p*=.015, Cramer’s V =0.23) but the residual chi-squared test did not show which valence was different between the two samples”.

**Discussion

It is very difficult to conclude that “the expression of anger in individuals with ASPD cannot be understood only as a simple basic emotion but could correspond to a more stable affective state like mood associated rather with highly saturated emotions” (p. 18 line 371-374), given the lack of information about the control group and the memories that each group recalled. Does the Self-Defining Memories task allow authors to make conclusions about "stable affective states" or just affective states during the task? The authors need to provide more information and control for important differences between the groups before this conclusion can be drawn.**

Answer: Thank you for your comment, we have further qualified our comments and rephrased our discussion.

**“Neutral state” (p. 19 line 403-404) should be re-written as “neutral expression” to avoid the inference that facial expression corresponds exactly to the participant’s emotional state.**

Answer: We have made corrections throughout the article.

**Minor**
**Please consider splitting up paragraphs based on topic in the introduction.**

Answer: Thank you for your feedback, we have made the changes in the document.

**Since the PCL-R was used, I would recommend using labels for the factors/facets that are typically used for this measure: Facet 1 “Interpersonal,” Facet 2 “Affective,” etc. (see work by Hare).**

Answer: Thank you for your feedback, we have made the changes in the document.

**“Mage” should be “M age” on p. 5 line 115.**

Answer: Thank you for your feedback, we have made the changes in the document.

**"Dichotomous fixe factor" should be changed to “dichotomous fixed factor” on p. 10 line 241.**

Answer: Thank you for your feedback, we have made the changes in the document.

**The authors should consider using person-first or similar language to refer to people in prison or forensic institutions. Instances of “inmate” should preferably be changed to “incarcerated person.” See this helpful resource from the Marshall Project (**[**https://www.themarshallproject.org/2021/04/12/what-words-we-use-and-avoid-when-covering-people-and-incarceration**](https://www.themarshallproject.org/2021/04/12/what-words-we-use-and-avoid-when-covering-people-and-incarceration)**)**

Answer: Thank you for your feedback, we have made the changes in the document.

Reviewer #2:

1) **Overall the paper would be improved by having some-one proofread it and improve the flow to make it easy to read and follow.**

Answer: Thank you for your feedback, the article has been proofread by a native English speaker.

2) **Title: I would suggest changing to: “Facial reactions to retrieving self-defining memories… or monitoring facial reactivity while asked to retrieve…”**

Answer: We have changed the title as you suggested: Monitoring emotional facial reactions of forensic inpatients with antisocial personality disorder during retrieval of self-defining memories

3) **Abstract:**
   a. **The first sentence in the introduction is awkward. What do the authors mean “very few studies have investigated its production”. Are the authors referring to naturally displaying facial expressions/producing facial expressions? They should also be specific. There are a number of studies examining facial reactivity/expressivity.**

Answer: We have modified the introduction. The following sentences have been added in the abstract: “While research has mainly focused on the ability to recognize emotional facial expressions and has identified difficulties in FE recognition in various mental and personality disorders [5–8], fewer studies have investigated the ability to produce emotional FE in personality disorders, especially in individuals with antisocial personality disorders (ASPD).”

b. **Similarly, in the abstract “during retrieval of autobiographical memories to be as close as possible to their personal feelings?” This is not clear and should be better phrased.**

Answer: We have made the following rewording in the abstract: “Rare studies have investigated how they produce their own emotional facial expressions. This study examines the production of facial emotional expressions of male inpatients with ASPD in a forensic hospital compared to a control group as they retrieve autobiographical memories. This design constitutes a specific ecological experimental approach fostering the evocation of personal feelings”.

c. **When the authors say they showed less neutral facial expression, does this mean they were more expressive? Again not clear. Also relates to later comment in my review.**

Answer: Thank you for your feedback, as you and others reviewers pointed out to us the way we referred to neutrality was unclear and confusing to the reader. We have reworded the neutral state throughout the article and the abstract.

In addition, these sentences were added to the discussion: “In addition, participants with ASPD showed fewer neutral FE than the control participants (Models 1 and 2). The typical narration of a memory includes phases of contextualization and presentation of the situation before describing the actual emotional event. In control participants, it is therefore consistent to observe mainly neutral expressions, which correspond to all these descriptive and non-emotional phases of memory. In participants with ASPD participants, expressions of anger dominated the overall narratives (see Figure 2). They thus expressed fewer neutral faces than the control participants.”

d. **Abbreviations (ASPD) in abstract need to be defined.**

Answer: Indeed, we have corrected this error in the abstract.

e. **The authors should be careful in their interpretation of the findings stating that “this study highlights the core role of anger in this psychopathology” –this is very general, the sample included individuals with APD so findings should be limited to APD.**

Answer: we have corrected this point

4) **Introduction**
   a. **P.4 There is a problem with authors argumentation. The first sentence argues that most studies in the area do not have ecological validity, and that their study examines spontaneous facial expression that arise when participants are asked to think of their emotional experiences. The problem with this is that the task they used had limitations which are outline below (next comment) and these are not mentioned in the limitations section.**

Answer: The SDM recall task is a task that has been proposed several times in autobiographical memory research. Self-defining memories are particular memories of autobiographical memory, emotionally more intense, directly related to identity construction, elaboration and achievement of life goals.

We have chosen this protocol because it allowed us to have the most open access possible to the life history of the patients and in particular to their important life memories. Thus, we have added the following sentences in the introduction: “Most studies about the emotional experiences of ASPD subjects have used experimental designs and their emotional responses were not studied from personal material but from image bases or external media. To our knowledge, no study has examined the spontaneous and personal emotional experiences arising from their own life history. The present study thus examined the emotional responses of participants with ASPD during the retrieval of self-defining memories (SDM) to mirror their own experiences as closely as possible.”

5) **Method**
   a. **There is a problem with the design on the task used. The authors used vague instructions asking participants to recall emotional events (which they then coded as positive, negative, mixed or neutral) vs asking them to recall a negative/positive/neutral memory. Looking at the data it is not clear that the task actually worked – did the participants actually follow the instructions or failed to do so due to bad memory/suppressed memory or simply because they did not want to understand or want to comply with instructions. This also raises concerns as figure 3 suggest that the task of recalling emotional events did not have the expected reaction for control participants (increase in happy expressions with the recall of positive events, or increase of sad angry or fearful facial expression in the event that a negative memory was recalled). This might be because of the high level of neutral expressions recorded by Facereader which is related to my next comment and suggestion about removing neutral expressions from the analysis.**

Answer: As mentioned earlier, we wanted the recall task to be as spontaneous as possible. We chose not to orient the valence of recalled memories so as not to introduce a bias in the emotional facial expressions. In addition, we chose to remove the neutral state from the dominance analyses, which allowed us to highlight that control individuals have a dominance of joy when recalling positive memories while antisocial individuals continue to have a dominance of their anger emotion.

6) **Results**
   a. **How original is this study and the findings presented? It is not surprising that individuals with APD are angry. Individuals with APD that end up in forensic settings obviously have anger issues. The data focused on fear are unexpected and interesting.**

Answer: Although the literature, particularly the clinical literature, mentions difficulties related to anger in this population, to our knowledge our study is the first to focus on their facial emotional reactivity during the recall of significant memories of their lives. Our results are interesting because they show that their autobiographical memory is specifically colored by the emotion of anger. It also encourages studies to better understand and manage anger that seems to be pathological in this disorder.

b. **The authors do not explain the findings well or in a context that the reader that is not familiar with how Facereader software operates, can be understood. The software by default categorizes the facial expression to neutral if it can not classify it as any other emotion. So what does it mean in relation the authors findings? It might be worth removing the neutral data and focus on the other emotions (happy, fear, sad, angry) to pick up any differences. It is hard to understand if the task participants were asked to performed “worked” as even when the memories were categorized as positive/negative the main expression produced was neutral. By removing the neutral data the authors might be able to pick up on more interesting findings.**

Answer: Thank you for your comments. We have chosen to retain the non-activation states and thus the prevalence of neutrality states in the analyses. Indeed, we still find this difference between the two populations interesting because it reinforces the place of reactivity associated with anger. However, we chose to further explain this neutrality state in the discussion section as follows: “In addition, participants with ASPD showed fewer neutral FE than the control participants (Models 1 and 2). The typical narration of a memory includes phases of contextualization and presentation of the situation before describing the actual emotional event. In control participants, it is therefore consistent to observe mainly neutral expressions, which correspond to all these descriptive and non-emotional phases of memory. In participants with ASPD participants, expressions of anger dominated the overall narratives (see Figure 2). They thus expressed fewer neutral faces than the control participants.

Moreover, we removed the neutral from the dominance analyses and obtained the following results: “Concerning Dominant FE in control participants, happy FE was expressed in 47.6% of SDM, angry FE in 27.6 %, sad FE in 13.3% and scared FE 11.4%) (χ^2^=16.57, *p<*.001, Cramer’s V =0.28). In individuals with ASPD, the Dominant FE was happy in 11.5% of SDM, angry in 75%, sad in 3.1% and scared FE in 10.4% (χ^2^=50.93, *p<*.001, Cramer’s V =0.52). There was a difference between the distributions of the two groups (χ^2^=50.24, *p<*.001, Cramer’s V =0.50). The residual chi-square test showed differences between the ASPD and the control samples for the happy FE (-3.36 vs. 3.21) and for the angry FE (3.42 vs. -3.27). Distributions of the dominant emotion according to group and valence of SDM are shown in Figure 3”.

Concerning the additive model of Dominant FE according to the group (Model 3), with the happy dominant FE as reference, the probability of observing the activation of angry dominant FE was higher for the ASPD group than for the control group. No main effect was observed for the other dominant FE (sad and scared). With the happy dominant FE and the neutral SDM as references, the probability of observing the activation of the angry dominant FE during the retrieval of positive SMD was greater for the APSD sample than for the control one (Model 4). Results of both models are shown in Table 1 and Model 4 is illustrated in Figure 3.”

c. **Figures 1-3 are not clear (font is small and fuzzy) making the figures hard to interpret. A suggestion would be to use color so it easier to differentiate between the emotions if this is not possible to organize them in a way that helps the reader (lighter color neutral, darker greys negatives then positives or something like that).**

Answer: Thanks for your feedback, we have improved the quality of the figures.

d. **Also there seems to be a large deviation between the control group and the APD group with regard to age and years of education. Were these looked into?**

Answer: Indeed, we have added clarifications concerning age and education between the two groups: “There was no significant age effect on the various facial expressions (neutral, happy, angry sad and scared) between the two groups. However, since most ASPD participants presented conduct disorders during childhood associated with school difficulties, we were not able to match education levels between APSD and control participants”.

e. **Minor point but table 2 and figures have different font than the rest of the document**.

Answer: We have corrected this point in table 2 and we have improved the quality of the figures and detailed the legends

7) **The discussion should be more focus on the explaining how the findings of the current study fit in the literature. Main finding seems to be that APD are angry but this is not new.**

Answer: We have reformulated our discussion to emphasize the innovative dimension of our study (study of the emotions associated with the retrieval of autobiographical memories with ASPD persons, and the innovative side of our experimental methodology and of our analysis method).

Reviewer #3

**Introduction**

**In my opinion, it would be more accurate to state that, to date, research has focused on the ability to recognize emotional facial expressions but not expressing them because I think there are more than few studies investigating the detection of FE in other individuals in participants with antisocial personality disorders. Of course, many of the existing studies did not investigate participants with a diagnosis of ASPD (which is a clear advantage of the current study) but instead antisocial or psychopathic personality traits/offenders/etc. However, I would like this statement of the authors to be more specific, especially since the authors do not only cite papers that focus on a sample with a diagnosis of ASPD**.

Answer : Thank you for your comment, we have reworded the introduction as follows: “While research has mainly focused on the ability to recognize emotional facial expressions and has identified difficulties in FE recognition in various mental and personality disorders [5–8], fewer studies have investigated the ability to produce emotional FE in personality disorders, especially in individuals with antisocial personality disorders (ASPD) [9,10]”.

**Second, I think the manuscript would benefit from a stronger differentiation between expressing emotions (e.g., anger), which can be objectively measured, and subjectively experiencing emotions.**

**In this context, I would like to ask, why the authors did not assess a subjective description of what the participants felt. Especially, since the instruction for SDM included choosing memories that elicit strong feelings and the use of the FaceReader instead of electromyography allows distinguishing between the different negative emotions.**

Answer: We have made this methodological choice because antisocial people have a deficit in the evaluation of their own feelings. Therefore we added this sentence and these references to the discussion: “However, asking people with APSD about their subjective feelings is particularly complex as they have a deficit in their appreciation of these [57,58]”.

**Further,** **I would like to suggest providing a more detailed explanation why particularly the expression of emotions is relevant in individuals with ASPD.**

Answer: We have added this point in the introduction: “The study of emotional expression in patients with ASPD is particularly important because it allows to examine the ability to communicate one's own emotional states and to explain possible difficulties in social interactions, especially in inducing fearful or aggressive responses. In these disorders, it is necessary to better articulate the person's subjective emotional experience and what they are able to communicate about it”.

**I would like to ask for a definition of “ecological setting”.**

Answer: We have corrected the presentation of the design “Most studies about the emotional experiences of ASPD subjects have used experimental designs and their emotional responses were not studied from personal material but from image bases or external media. To our knowledge, no study has examined the spontaneous and personal emotional experiences arising from their own life history.”

**As psychopathy and its factors/facets are analyzed, in my opinion, the construct of psychopathy and the PCL-R should be described in more detail.**

Answer: Thank you for your comment. As mentioned by the first reviewer and following your feedback, we have decided to delete these analyses, as we question the statistical power of these results given the size of our ASPD sample. In addition, we believe that this part led to confusion in the concepts (ASPD, psychopathy) for the reader. We chose to keep PCL-R scores in our sample only for descriptive purposes in the methods.

**In my view, the manuscript would benefit from a clear statement with regard to the hypotheses. (Exploratory analyses should be labeled as such if necessary.)**

Answer: we have reformulated our hypotheses in the introduction as follows: “We hypothesize that individuals with ASPD produce more anger-associated FE than healthy participants during the retrieval of SDM. We also examined how participants with APSD expressed other emotions during memory recall (happy, sad and scared) in comparison with control participants, and quantified the part devoted to the non-expression of emotional FE (neutral states).

**Methods**

**How was ensured that that the control participants had no mental or behavioral difficulties? How were they recruited?**

Answer: Indeed, we have corrected this error and have provided information regarding the recruitment of our control population. The following sentences has been added in the methods: “The control sample was recruited through an advertisement distributed on social networks and from the staff of the universities of Lille in France and Mons in Belgium”.

**It seems like education level and age might differ between groups. I think the authors should comment on that in their manuscript.**

Answer: we have added a sentence to explain why we were unable to match on education level in the methods “There was no significant age effect on the various facial expressions (neutral, happy, angry sad and scared) between the two groups. However, since most ASPD participants presented conduct disorders during childhood associated with school difficulties, we were not able to match education levels between APSD and control participants”.

**How was the neutral video fragment for calibration selected?**

Answer: As you point out, we have not provided an explanation for this and have corrected it with the following sentence: “This neutral video fragment was captured during the test phase described above”.

**Results**

**What was the minimum number of SDM per participant after the exclusion?**

Answer: We have provided this information in the result section: “Furthermore, since five ASPD participants moved during the retrieval of one of their five SDM, FaceReader was unable to analyze FE during retrieval, thus reducing the number of SDM in this group to 96”.

**At first, I was confused by the structure of the statistical analysis section and the results. In my opinion, the results regarding the valence of the SDM and the associated chi squared test should be reported in the beginning, because this is essential for the interpretation of the results (the test is mentioned in the second paragraph of the methods but the results are reported in the third paragraph of the results section).**

Answer: Indeed, thank you for pointing out this error. We have reorganized the results to make them more consistent.

**Figure 1: Information regarding the boxplots might help the reader to better understand the results (e.g., does the boxplot show the mean or the median?)**

Answer: we have unpacked the figure captions to provide more clarity.

**I would be interested in the estimate for the main effect of valence in Model 2.**

Answer: Model 2 is a multiplicative model and if the software gives us indeed, the main effects these are not interpretable. We have chosen not to add these results because we think that they could be confusing for the reader. In addition, we really want to focus our research on antisocial personality disorder. Also, we chose to remove the main effects of valence in Model 1 and Model 3. We feel that these results were confusing for the reader.

**I think figure 3 is not referenced in the text.**

Answer: Indeed, thank you for your comment; we have corrected it in the article.

**I understand that in Table 1 shows only the interaction effects for model 4 but not the estimates that are reported for model 3. Why? Are there differences?**

Answer: As for your previous comment, model 4 is a mutiplicative model, so the main effects provided by the software are not interpretable and could have led to confusion. Furthermore, the estimates of model 3 have been corrected following the removal of neutrality in dominance and appear in table 1.

**The naming of the factors and subscales of the PCL-R should be consistent in the table and the text.**

Answer: As mentioned earlier, we have chosen to remove the analyses and correlation table from the article because we felt that these analyses were not powerful enough given our ASPD sample size and also because it confounded psychopathy and antisociality.

**Discussion**

**I think the conclusion that participants with ASPD used fewer FE associated with the neutral state than the control participants independent of the valence of the SDM should not be based on model 1, since this model did not include the valence.**

Answer: we have corrected these points in the discussion.

**The discussion of the results with regard to psychopathy is mainly focused on the facet 3 and 4. However, in the introduction it seemed like an aim of the study to investigate if experiencing fewer emotions is related to callous-unemotional traits, which should also be represented by facet 2**.

Answer: We have chosen to remove these analyses; we also removed this part in the discussion.

**I think the limitation that the degree of congruence between FE and subjectively experienced feelings was not measured is a major limitation and I think the manuscript would benefit from a more detailed discussion of this issue and its relevance for the conclusion than can be drawn based on this study.**

Answer: As mentioned earlier, it is very difficult to collect the subjective emotional feelings of people with ASPD. However, according to your comment, we have added the following sentence in the limit section of the discussion: “Moreover, we did not measure the degree of congruence between FE and feelings, a step that would be important for a deeper understanding of emotions in the field of psychopathy (Kosson, personal communication). However, asking people with APSD about their subjective feelings is particularly complex as they have a deficit in their appreciation of these [57,58]”.

**Finally, I am wondering if the stronger expression of anger during retrieval of SDM in individuals with ASPD can be generalized to everyday situations and can be interpreted as chronic anger. This concern is strengthened by the fact that SDM in individuals with ASPD might be more traumatic and anger evoking that the SDM of control participants. Since only the general valence of the SDM was considered but not the related emotion or the intensity, in my view, it might still be possible that group differences in anger expression are based on differences in SDM.**

Answer: In this sense, we have added these elements in the discussion of the article: Moreover, it will be interesting to complement the measures of emotional facial reactivity made from self-defining memories with analyses of memories to emotional situations of daily life, especially because of the particular characteristics of SDM of incarcerated patients. This type of study will help confirm whether anger hyper-expressivity in patients with ASPD is specific to significant personal past episodes or is generalizable to all emotional situations.
