# Supplementary material for: Monitoring the emotional facial reactions of individuals with antisocial personality disorder during the retrieval of self-defining memories
Source: PLoS One. 2022 Jun 8;17(6):e0268818. doi: 10.1371/journal.pone.0268818 (PMC9176833; doi:10.1371/journal.pone.0268818)
Supplement: Attachment — Submitted filename: 2nd review.docx [file pone.0268818.s005.docx]

Thank you for inviting me to review a revision on this manuscript which presents a study that examines the facial reactivity during the retrieval of self-defining memories in individuals with Antisocial Personality Disorder (APD) and controls.

Unfortunately there are still a number of weaknesses that affect my enthusiasm for the manuscript with the more pronounced one being the language and syntax errors. Overall the paper would be improved by having an English speaker proofread the paper to improve it and make it easy to read and follow. There are still a lot of errors in the revised manuscript submitted.

a. For example the title should be: “Monitoring the emotional facial reactions of individuals with antisocial personality disorder during the retrieval of self-defining memories”
b. Addressing these errors in the abstract and the main paper would greatly enhance the paper. There are many so I will not address them in the review but these will be picked up by a native English speaker.
