# Supplementary material for: Monitoring the emotional facial reactions of individuals with antisocial personality disorder during the retrieval of self-defining memories
Source: PLoS One. 2022 Jun 8;17(6):e0268818. doi: 10.1371/journal.pone.0268818 (PMC9176833; doi:10.1371/journal.pone.0268818)
Supplement: Attachment — Submitted filename: Response to Reviewers LAST VERSION.docx [file pone.0268818.s006.docx]

**Reviewer #1:** This study adds to just a few others that have examined the production (rather than recognition) of facial expressions in people with antisocial personality disorder (ASPD). The authors have addressed several of the concerns I noted in my previous review. However, some of my concerns remain. I hope the authors can address the points below to ensure that the contribution of their data to our understanding of ASPD is clear.

My main concern is that the authors have concluded from their data that “anger is a core emotion” (lines 459-460) of ASPD. Unfortunately the study design does not afford this broad conclusion. My conclusion from their data is this: when asked to describe important life events, people with ASPD are less likely to describe positive events and more likely to make facial expressions associated with anger.

Answer: Thank you for your comment. We have modified the conclusion to more focus on the facial activation and not on the emotional experience properly. About the less proportion of positive events in the participants with ASPD, statistical analyses did not allowed concluding to significant valence difference between our two populations.

Introduction

I appreciate that the authors added to their list of hypotheses. However, the last sentence in the paragraph is unclear (lines 114-116). What were the authors’ hypotheses regarding happy, sad, and scared expressions? Also, the authors note in this sentence that they “quantified the part devoted to the non-expression of emotional FE.” How is that related to the authors’ hypotheses?

Answer: Indeed, we have reworded the hypothesis part of the introduction to clarify it: *“Additionally, we expected that this overactivation of anger-associated FEs may lead to an underactivation of the other emotional or nonemotional FEs (happiness, sadness, fear and neutral) ».*

Methods

The authors have added necessary information about the control group. Now, the authors should show that education level was unrelated to frequency of facial expressions (especially neutral and angry expressions). Since the ASPD and control group differed in education level, and the control group was even recruited from university staff, it is possible that education level is driving the significant differences between groups. In fact, education level is probably not the only socioeconomic factor that differs between ASPD group, which was recruited from a forensic hospital, and the control group, recruited from university staff. Ideally, the authors would note this in the limitations section.

Answer: Indeed, we have made statistical analyses and we have not found an effect of the education level in the activation or non-activation of the action units (see comment for the reviewer 3).

In my previous review I requested that the authors distinguish between ASPD and psychopathy, because they reported findings related to both constructs. Since the authors no longer report analyses of psychopathy in the manuscript, it shouldn’t be necessary to describe the psychopathy measurement in the Methods section.

Answer: In agreement with the other reviewers, we have decided to retain the clinical description for psychopathic traits. We believe that a comprehensive description of our clinical population can be useful to the scientific and medical community.

The authors’ rationale for Bayesian analyses is still unclear to me. What metric did the authors use to determine that Bayesian analyses were the best statistical method? Power analysis? Can the authors point me to a paper showing how to decide between Bayesian analyses and the general linear model considering the sample size and experimental design? I’m afraid the rationale is too vague. My worry is that readers may wonder why the authors chose Bayesian analyses for data that would typically have been analyzed with the general linear model, and that readers may think the authors chose the analyses that supported their hypotheses. Clarifying the rationale for the analyses would help!

Answer: Thank you for your comment. We have added the following sentences in the statistical analyses section: *" Statistical analyses were performed using Bayesian methods, on the one hand because of the experimental design and on the other because it is commonly used in autobiographical studies [40–42]."*

*« If the 95% CI does not include the zero value, then we can reject the null hypothesis and consider the estimator to be most likely different from zero and to reflect a decrease or increase in the probability as a function of the observed sign ».*

Results

In my previous review I asked whether the ASPD group described more negative life events in the SDM task. I thank the authors for clarifying that “Chi-squared tests demonstrated a difference between the two groups but the residual chi-squared test did not show which valence was different between the two samples” (lines 345-347). This means there was a difference between groups, correct? The raw percentages suggest the groups differed in rates of positive stories (ASPD = 22.8%, control = 36.2%) and neutral stories (ASPD = 34.7%, control = 16.2%). I think it would be important to acknowledge these differences, because they may have affected the facial expressions the ASPD group made while describing life events.

Answer: Although we could observe differences between the proportions of the different valences between our two groups, the statistical analyses did not allow us to conclude that there were significant differences. In addition, we completed the text to improve the understanding of the data and the statistical methodology: *« Chi-squared tests demonstrated a difference between the two groups (χ^2^ = 10.46, p = .015, Cramer’s V = 0.23). As the valence table corresponded to a 4*2 Table (4 valences and 2 groups), it was necessary to perform a residual chi-squared test to discriminate which valence differed between our two groups [48]. However, the residual chi-squared test failed to exceed the +/- 2 criteria and in this way, does not allow us to conclude that there is a significant difference in the production of memories depending on the valence between the ASPD and the control groups ».*

FaceReader detected fewer neutral expressions in the ASPD group. Wouldn’t one interpretation of the data be that the ASPD group uses more facial expressions when describing life events? Is that surprising, given the ASPD group were describing more neutral life events? Conversely, it sounds like the more educated control group described more positive life events but made fewer facial expressions when describing those events.

Answer: Thank you for your comment. To answer the first question, indeed, participants with ASPD have more emotional EFs and in particular those associated with anger than controls. To answer your other question, as we explained above, there is no statistical difference between the valences of the SDMs of our two populations. In this way, and in order not to over-interpret our data, we cannot conclude as you suggest.

Discussion

Related to my point above, the data do not support this statement: “ASPD participants retrieved as many SDM of each valence as the control sample” (lines 387-388). Please revise, as the groups differed in the rates of positive and neutral stories.

Answer: Indeed, we have modified the discussion part as follow:  *“This activation of the angry FE cannot be explained by the emotional content of the SDMs retrieved by ASPD participants because we did not observe a significant difference in terms of valence between our two groups ».*

I appreciate that the authors now cite Barrett et al. (2019). However, note that Barrett et al.’s comprehensive review found that facial muscle movement does not reliably express any one emotion category. Based on that finding, what do the current facial muscle data tell us about the subjective feeling states of people with ASPD during this task? Should we assume that “angry expressions” signal an underlying subjective feeling of “anger”? This limitation should be noted. I appreciate that the authors already highlight the need for measuring participants’ subjective feelings during the task.

Answer: Thank you for your comment. We have added the following sentences in the limitations part of the discussion: *“Moreover, we did not measure the degree of congruence between FE and feelings, which should be interesting in future study projects. This comparison between facial muscle activation and subjective feeling would be important for a deeper understanding of emotions in the field of psychopathy (Kosson, personal communication).* *However, asking people with APSD about their subjective feelings is particularly complex, as they have a deficit in identifying and understanding them [52,53]”*

Minor

Again, I would advise changing “neutral state” (line 223) to “neutral expression” to avoid the inference that facial expression corresponds exactly to a person’s emotional state.

Answer: We have modified all the “neutral state” by “neutral expression”.

Please revise “consider the estimator to be truly different” (line 313) to “consider the estimator to be most likely different.”

Answer: We have modified the text as you propose.

I found this statement unclear: “asking people with APSD about their subjective feelings is particularly complex as they have a deficit in their appreciation of these” (lines 440-441). The phrase “appreciation of subjective feelings” is vague. Please revise.

Answer: Thank you for your comment. We have modified the sentence as follow: *“However, asking people with APSD about their subjective feelings is particularly complex, as they have a deficit in identifying and understanding them [52,53]”*

I would recommend revising this conclusion: “our findings shed new light on the emotional experience” (line 457). See my comments above about what facial muscle movements tell us about a person’s emotional experience.

Answer: Thank you for your comment. We have modified the sentence as follow: *« This study highlights the core activation of the FE associated with the emotion of anger in a forensic population with ASPD during the retrieval of significant memories of their past. This particular activation associated with SDMs could have a role in the construction and perhaps the maintenance of ASPD and requires special attention and a specific psychological accompaniment”.*

**Reviewer #2:** Thank you for inviting me to review a revision on this manuscript which presents a study that examines the facial reactivity during the retrieval of self-defining memories in individuals with Antisocial Personality Disorder (APD) and controls.
Unfortunately there are still a number of weaknesses that affect my enthusiasm for the manuscript with the more pronounced one being the language and syntax errors. Overall the paper would be improved by having an English speaker proofread the paper to improve it and make it easy to read and follow. There are still a lot of errors in the revised manuscript submitted.
a. For example the title should be: “Monitoring the emotional facial reactions of individuals with antisocial personality disorder during the retrieval of self-defining memories”
b. Addressing these errors in the abstract and the main paper would greatly enhance the paper. There are many so I will not address them in the review but these will be picked up by a native English speaker.

Answer: Thank you for your comment, we have modified the title as you propose and we have sent our article to “American Journal experts” to improve the language and the syntax of our manuscript.

We join a certificate attesting the rewieving perfomed. This certificate was issued on February 16, 2022 and may be verified on the AJE website using the verification code 428B-0326-2E11-77AE-9F05.

**Reviewer #3:** In my view, the authors have done a good job revising the paper and I think the paper clearly improved. My only remaining concern is related to the clarification in the methods section and my previous comment on the description of the samples. In my opinion, differences in age and education (even if neglectable and not critical for the main results) should be mentioned as a possible limitation. Further, if ASPD or other mental disorders have not been assessed in the control sample, this should be stated and mentioned as a potential limitation.

Answer: Thank you for your comment. As you pointed out concerning the education level, we did further statistical analyses and observed that there was no effect of education level on the activation or non-activation of our participants' action units:

Neutral~Age: Estimate = 0.01, 95%CI = [-0.01; 0.04]), Neutral~Education level: Estimate = 0.06, 95%CI = [-0.03; 0.14];

Happy~Age: Estimate = 0.01, 95%CI = [-0.03; 0.05]), Happy~Education level: Estimate = 0.05, 95%CI = [-0.11; 0.19];

Angry~Age: Estimate = -0.02, 95%CI = [-0.05; 0.02]), Angry~Education level: Estimate = -0.15, 95%CI = [-0.30; 0.00].

Furthermore, to answer your comment about our control sample, it was composed of people from the general population for whom the exclusion criteria for participation in the research were to have a psychiatric diagnosis of axis 1 of the DSM.
