# Supplementary material for: Monitoring the emotional facial reactions of individuals with antisocial personality disorder during the retrieval of self-defining memories
Source: PLoS One. 2022 Jun 8;17(6):e0268818. doi: 10.1371/journal.pone.0268818 (PMC9176833; doi:10.1371/journal.pone.0268818)
Supplement: Attachment — Submitted filename: response to reviewers.docx [file pone.0268818.s007.docx]

Reviewer #1: The authors have addressed my previous concerns, although I'm still unsure about one issue regarding the valence of SDMs. The authors performed a chi-squared test that showed that ASPD and control participants differed in the valence of memories (lines 348-349). The follow-up residual chi-squared tests did not reveal which valence differed between the two groups. So the groups differed, but the authors cannot conclude how they differed. Yet the authors state that the data "does not allow us to conclude that there is a significant difference in the production of memories depending on valence" (lines 352-353). That statement seems to inaccurately describe the first chi-squared test. Wouldn't it be fairer to say the data "does not allow us to conclude which valence of SDMs was produced more frequently by either group"? I'm willing to accept that the authors are simply interpreting the data very cautiously.

Response: We integrated the sentence proposed by the reviewer : *« the data does not allow us to conclude which valence of SDMs was produced more frequently by either group ».*

Reviewer #2: At the very least, I'd recommend the authors revise the following statement in the discussion (lines 436-437): "it appears that anger notably associated with aversive life events should be the target of psychologic interventions." How can the authors make conclusions about aversive life events if the groups did not differ in terms of SDM valence (above)? Also, the data seem to show that the ASPD group displayed more facial expressions associated with anger while talking about positive memories but not negative or mixed memories (Model 4 in Table 1). I think the sentence in lines 436-437 would be fine if it removed the phrase "associated with aversive life events."

Response: *We decided to delete the part of the sentence: « associated with aversive life events ».*
